# Supplementary material for: How often do leading biomedical journals use statistical experts to evaluate statistical methods? The results of a survey
Source: PLoS One. 2020 Oct 1;15(10):e0239598. doi: 10.1371/journal.pone.0239598 (PMC7529205; doi:10.1371/journal.pone.0239598)
Supplement: S1 Table — (DOCX) [file pone.0239598.s001.docx]

**S1 Table. Number of survey responses by subject area (Web of Science sub-discipline categories for the discipline of biomedicine).**

# Biomedicine sub-domains

| field | n |
| --- | --- |
| Cardiac Cardiovascular Systems | 6 |
| Emergency Medicine | 5 |
| Nursing | 4 |
| Otorhinolaryngology | 4 |
| Pediatrics | 4 |
| Surgery | 4 |
| Biology | 3 |
| Gastroenterology Hepatology | 3 |
| Health Care Sciences Services | 3 |
| Health Policy Services | 3 |
| Infectious Diseases | 3 |
| Medicine General Internal | 3 |
| Rehabilitation | 3 |
| Urology Nephrology | 3 |
| Allergy | 2 |
| Audiology | 2 |
| Cell Tissue Engineering | 2 |
| Clinical Neurology | 2 |
| Dentistry Oral Surgery Medicine | 2 |
| Dermatology | 2 |
| Medical Informatics | 2 |
| Multidisciplinary Sciences | 2 |
| Neuroimaging | 2 |
| Nutrition Dietetics | 2 |
| Obstetrics Gynecology | 2 |
| Opthalmology | 2 |
| Orthopedics | 2 |
| Pathology | 2 |
| Pharmacology Pharmacy | 2 |
| Public Environmental Occupational Health | 2 |
| Rheumatology | 2 |
| Substance Abuse | 2 |
| Toxicology | 2 |
| Andrology | 1 |
| Anesthesiology | 1 |
| Biophysics | 1 |
| Biotechnology Applied Microbiology | 1 |
| Critical Care Medicine | 1 |
| Endocrinology Metabolism | 1 |
| Ergonomics | 1 |
| Geriatrics Gerontology | 1 |
| Gerontology | 1 |
| Hematology | 1 |
| Integrative Complementary Medicine | 1 |
| Parasitology | 1 |
| Primary Health Care | 1 |
| Psychiatry | 1 |
| Radiology Nuclear Medicine Medical Imaging | 1 |
| Respiratory System | 1 |
| Sports Sciences | 1 |
| Transplantation | 1 |
