# Supplementary material for: How often do leading biomedical journals use statistical experts to evaluate statistical methods? The results of a survey
Source: PLoS One. 2020 Oct 1;15(10):e0239598. doi: 10.1371/journal.pone.0239598 (PMC7529205; doi:10.1371/journal.pone.0239598)
Supplement: S1 Fig — The dashed line indicates the < = 10% cut-off point whereby statistical review was considered ‘rare’ and respondents were re-directed towards the end of the survey (see methods section for details). (DOCX) [file pone.0239598.s002.docx]

# Frequency of statistical review


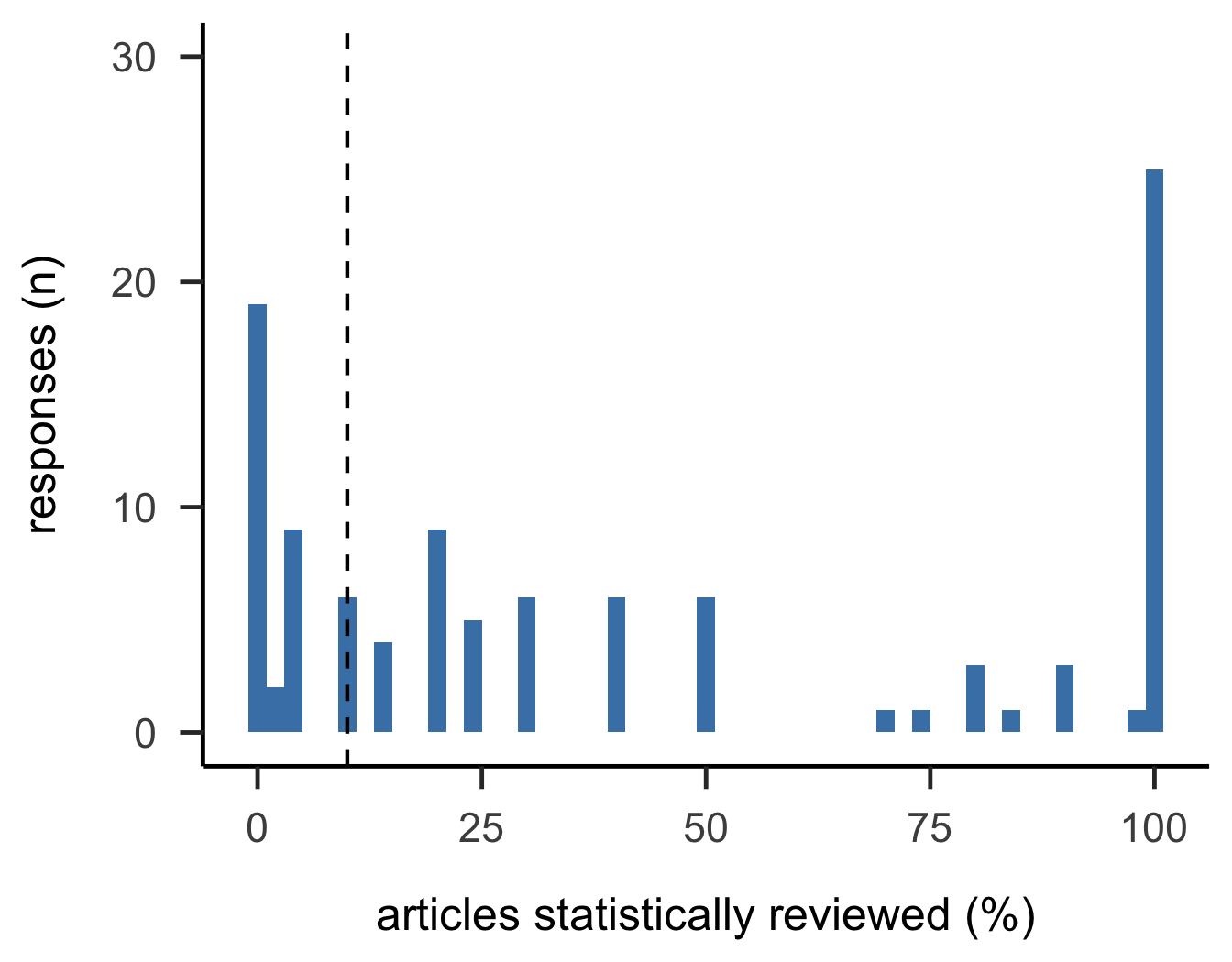


**S1 Fig. Histogram showing distribution of estimates for the percentage of original quantitative research articles that undergo statistical review.** The dashed line indicates the <= 10% cut-off point whereby statistical review was considered ‘rare’ and respondents were re-directed towards the end of the survey (see methods section for details).
