# Supplementary material for: How often do leading biomedical journals use statistical experts to evaluate statistical methods? The results of a survey
Source: PLoS One. 2020 Oct 1;15(10):e0239598. doi: 10.1371/journal.pone.0239598 (PMC7529205; doi:10.1371/journal.pone.0239598)
Supplement: Attachment — Submitted filename: Response to reviewers.docx [file pone.0239598.s003.docx]

**Memo to**: PLoS One editors

**From**: Steven Goodman

**Re**: Response to reviewers

**Manuscript**: PONE-D-20-12128
**Title**: How often do leading biomedical journals use statistical experts to evaluate statistical methods? The results of a survey.

Editors Comments:

1.) “In order to improve reproducibility and replicability, please provide the list of journals surveyed. Please also provide a statement about whether your sample is representative of a larger population.”

We have provided a complete anonymized datafile, with the computational environment, in the OSF framework, as indicated in the Open Science statement of the paper. However, our IRB approved consent form explicitly stated that we would not identify the journals who answered the survey. We described in detail the sampling frame in lines 59-64:

*“From the complete list of Web of Science subject categories (228) we identified all 68 sub-domains representing biomedicine. We selected the top 5 journals by impact factor within each sub-domain. We supplemented this list with 68 additional journals previously included in the survey by Goodman and colleagues [22], and assigned each of these to their relevant sub-domain. Finally, we removed any duplicates that appeared in multiple sub-domains. This resulted in a sample of 364 journals.”*

S file Senate very jazz but everything very engagedupplementary Table 1, included in the manuscript file shows the number of responding journals in each specialty and sub-specialty. This provides the best evidence we can provide relative to the representativeness of the achieved sample. We do not assert that this is perfectly representative of the whole sample; in fact, as we discuss at length in the manuscript, we believe that the sample is likely to be biased towards more statistical review. This only strengthens the findings, because the use of statistical review in this sample is rather low.

2.) Thank you for stating the following in the Funding Statement Section of your manuscript:

"This work was enabled in part by a fellowship grant to the Meta-Research Innovation Center at Stanford (METRICS) from the Laura and John Arnold Foundation. The Meta-Research Innovation Center Berlin (METRIC-B) is supported by a grant from the Einstein Foundation and Stiftung Charité."

We note that you have provided funding information that is not currently declared in your Funding Statement. However, funding information should not appear in the Acknowledgments section or other areas of your manuscript. We will only publish funding information present in the Funding Statement section of the online submission form.

Please remove any funding-related text from the manuscript and let us know how you would like to update your Funding Statement. Currently, your Funding Statement reads as follows:

"The author(s) received no specific funding for this work."

Done.

**Reviewer #1**: The authors conducted a survey to explore how often high impact factor journals in all clinical fields seek statistical expertise for the papers they publish, reasons for not doing so etc. The topic is of general interest and the paper is easy to read.

I am afraid I have not many things to say to improve the paper. There are many issues that are addressed to a good extent….

The reviewer’s comments are appreciated. S/he provides no comments requiring reply or modification of the paper.

**Reviewer #2**: I read with great interest they survey by Hardwicke and Goodman entitled "How often do leading biomedical journals use statistical experts to evaluate statistical methods? The results of a survey.". The manuscript is well written and the findings are clearly presented. I have the following 2 comments:

1) I am wondering whether the authors could namely specify the 107 journals in Supplementary Table 1.

For the reasons provided in the response to the editor's comments, we unfortunately cannot do this; the journals were promised confidentiality. mood

2) Considering the journals included in the current and the initial survey 20 years ago, it would be interesting to see graphically the proportion of changes for specific "variables".

Unfortunately, we no longer have the data files from the survey 20 years ago. We would have liked to have been able to conduct the same analysis, but we couldn't.
